# Water exchange colonoscopy decreased adenoma miss rates compared with literature data and local data with CO_2_ insufflation: an observational study

**DOI:** 10.1186/s12876-019-1065-2

**Published:** 2019-08-14

**Authors:** Chi-Liang Cheng, Yen-Lin Kuo, Yu-Hsi Hsieh, Jui-Hsiang Tang, Felix W. Leung

**Affiliations:** 1 Division of Gastroenterology, Department of Medicine, Evergreen General Hospital, 150 Huan-Zhong East Rd., Zhongli District, Taoyuan, 320 Taiwan; 2 Division of Gastroenterology, Dalin Tzu Chi Hospital, Buddhist Tzu Chi Medical Foundation, 2 Minsheng Rd., Dalin Township, Chiayi County, 622 Taiwan; 3 Division of Gastroenterology and Hepatology, Department of Internal Medicine, School of Medicine, College of Medicine, Taipei Medical University, 252 Wuxing St, Xinyi District, Taipei, 100 Taiwan; 4 Division of Gastroenterology, Department of Medicine, Sepulveda Ambulatory Care Center, Veterans Affairs Greater Los Angeles Healthcare System and David Geffen School of Medicine at UCLA, 16111 Plummer St, North Hills, CA 91413 USA

**Keywords:** Colonoscopy, Water exchange, Carbon dioxide, Adenoma miss rate, Colon polyp

## Abstract

**Background:**

Reports showed adenoma miss rates (AMRs) of 22.5–27% in the right colon and 23.4–33.3% in the proximal colon. Missed lesions could contribute to postcolonoscopy cancers. Water exchange (WE) with near-complete removal of infused water during insertion increased adenoma detection rate but the impact on AMR had not been reported. We hypothesized that WE could reduce AMRs. Study 1 compared the AMRs of WE with literature data. Study 2 developed local AMR data with CO_2_ insufflation.

**Methods:**

The lead author attended a research seminar in 2017 on WE colonoscopy. For performance improvement, study 1 was undertaken. When data in study 1 confirmed WE produced a considerably lower AMRs in the right and proximal colon, study 2 with CO_2_ insufflation was performed.

**Results:**

Eighty-six patients completed each study. In study 1, WE removed 89% of infused water upon arrival to the cecum. The AMRs of right colon (17.5%) and proximal colon (15.5%) were considerably lower than those in the literature. Upon completion of study 2, compared with local data of CO_2_ insufflation, WE showed a significantly lower AMR in the right (17.5% vs. 33.8%, *P* = 0.034) and proximal (15.5% vs. 30.4%, *P* = 0.018) colon, respectively. The major limitation was that the investigation consisted of two consecutive observational studies, not a randomized controlled trial (RCT).

**Conclusions:**

WE with near-complete (89%) removal of infused water during insertion significantly decreased AMRs in the right and proximal colon compared with literature data and those of CO_2_ insufflation in our hands. The provocative data warrant confirmation in a RCT.

**Trial registration:**

NCT03832322 (Retrospectively registered on February 2, 2019).

## Background

Colonoscopy is widely practiced, generally safe, accurate for detecting and prevention of colorectal cancer (CRC) [1], but is not perfect. A substantial number of neoplastic lesions are missed, according to tandem air insufflation colonoscopy studies [2–6]. The adenoma miss rates (AMRs) in the right and proximal colon ranged from 22.5 to 27% and 23.4 to 33.3%, respectively [2–6]. It has been reported that 58% of postcolonoscopy CRCs (PCCRCs) were attributed to missed lesions [7]. PCCRCs were 2.4 times more likely to arise in the proximal than in the distal colon [8]. Inadequate bowel preparation, lesions located behind folds or having a nonpolypoid shape, incomplete colonoscopy, a short withdrawal time, and suboptimal inspection techniques contributed to missed neoplasias [9, 10]. Small sessile polyps with advanced histology are more common in the proximal colon and are easily missed [11, 12]. For performance improvement, approaches that increase adenoma detection rate (ADR) conceivably could reduce AMR.

Water exchange (WE) colonoscopy is characterized by gasless insertion into the cecum in clear water and has been shown to significantly reduce insertion pain, facilitate the completion of colonoscopy and significantly increase bowel cleanliness, even in the right colon [13, 14]. WE with near-complete removal of infused water during insertion improved the overall ADR [13–17], right and proximal colon ADRs [14–18] compared with those of air insufflation. The impact of WE on AMR, however, had not been reported.

The lead author attended a research seminar in 2017 on WE colonoscopy. After mastering the basic skills of WE, the first performance improvement study (study 1) pertained to collection of data on WE AMRs in the right and proximal colon compared with those reported in the literature. When WE showed considerably lower AMRs, the second observational study (study 2) evaluated the corresponding AMRs with CO_2_ insufflation. The two studies demonstrated how WE could fit into the goal of performance improvement in our practice setting. The data in these two consecutive studies were compared to determine if our local data could show significant AMR differences to warrant implementation of a randomized controlled trial (RCT).

## Methods

This was a retrospective observational study. Consecutive patients of tandem colonoscopy were enrolled in these two performance improvement studies from July to November 2018 at Evergreen General Hospital, Taoyuan, Taiwan. Written informed consent was obtained from all participating patients for tandem colonoscopy. The Joint Institutional Review Board of Taiwan approved report of the prospectively collected AMR data (for 172 patients).

### Participants

Consecutive patients aged 20 years or older undergoing colonoscopy for screening and surveillance were eligible for enrollment. Because WE needed longer colonoscopy examination time compared with CO_2_ insufflation, we limited enrollment of same-day repeated examinations during study 1 to no more than 2 within 1 day in order to minimize the burden on the endoscopy suite. The criteria for exclusion from data analysis included single same-day colonoscopy, familial adenomatous polyposis and hereditary nonpolyposis CRC syndrome, a personal history of inflammatory bowel disease, previous colonic resection, inability to achieve cecal intubation, colonic obstruction, poor preparation, inability to completely remove polyps, gastrointestinal bleeding, American Society of Anesthesiology classification of physical status ≥3, mental retardation, pregnancy, and refusal to provide informed consent.

### Bowel preparation and sedation

All patients received a split dose of 3-L polyethylene glycol (Klean-Prep, Helsinn Birex Pharmaceuticals, Ltd., Dublin, Ireland) for bowel preparation. Colonoscopy was performed with moderate sedation (intravenous fentanyl plus midazolam) administered by the colonoscopist.

### First-pass colonoscopy

Colonoscopies were performed by two endoscopists (CL Cheng and YL Kuo) using a standard colonoscope (CF-Q260AL/I; Olympus, Tokyo, Japan). If same-day bidirectional endoscopy was arranged, esophagogastroduodenoscopy was performed first. Colonoscopy began with the patients in the left lateral position. In study 1 (the WE group), the air pump was turned off and the colon was irrigated with water during insertion. WE entailed the infusion of water to open the lumen and sequential suction of water. When the cecum was reached and after most of the water was suctioned, the CO_2_ was opened. In study 2 (the CO_2_ group), colonoscopy was performed with minimal insufflation to aid insertion. Cleaning in the CO_2_ group was performed during withdrawal. Cecal intubation was defined as the passage of the scope tip beyond the ileocecal valve with visualization of the ileocecal valve and appendix orifice.

Polyp resection was performed during insertion and withdrawal. All proximal polyps were removed irrespective of size and appearance. All diminutive polyps with hyperplastic appearance (based on narrow band imaging) in the rectosigmoid colon were documented and left alone. Polyp size was determined by comparison with opened colonoscopic biopsy forceps pushed against the polyp.

The following information was recorded: bowel preparation quality using the Boston Bowel Preparation Scale (BBPS) score [19]; the doses of sedative medications; the amount of water infused and suctioned during insertion and withdrawal; the procedure time required for insertion and withdrawal; the need for abdominal compression and/or position change to assist insertion; and the polyp number, size, histology and location.

### Second-pass colonoscopy

After the first complete withdrawal of the colonoscope, the same endoscopist performed a second examination aided by CO_2_ insufflation in both study 1 and study 2. The colonoscope was reinserted into the cecum, and the entire colon was re-examined. Polyp resection was performed during insertion and withdrawal. The following information was recorded: the amount of additional sedative medications; the procedure time required for insertion and withdrawal; and the missed polyp number, size, histology and location.

### Definition

Complete colonoscopy was defined as successful cecal intubation. The time between scope insertion and cecal intubation was insertion time. Time from cecal intubation to colonoscope withdrawn from the anus was the withdrawal time (included time required for cleaning and treatment). The total procedure time was the sum of the insertion and withdrawal times. A total BBPS score of 5 or less represented poor colon preparation.

The right colon included the cecum, ascending colon, and hepatic flexure; the proximal colon included the right and transverse colon. Adenomas included all adenomas and sessile serrated adenomas. Adenomas with a diameter ≥ 10 mm, a (tubulo) villous structure, or high grade dysplasia were classified as advanced adenomas.

Lesions detected on the second-pass examination were considered missed in the first-pass, with the exception of the diminutive polyps in the rectosigmoid colon that remained after the first-pass colonoscopy. Lesions detected on the first-pass examination were used to calculate ADR and advanced ADR, the proportion of colonoscopies with at least one adenoma or one advanced adenoma, respectively. Mean adenoma per procedure (MAP) was measured by the total number of adenomas detected divided by the number of colonoscopies. The proximal hyperplastic polyp detection rate (PHP-DR) was the proportion of patients undergoing colonoscopy for which at least one hyperplastic polyp was identified in the proximal colon.

The miss rate per participant was the proportion of participants with at least one adenoma or polyp missed in the first-pass examination. The AMR and hyperplastic polyp miss rate (HPMR) were calculated as the number of adenomas and hyperplastic polyps missed in the first colonoscopy divided by the total number of adenomas and hyperplastic polyps detected during both the first and second colonoscopies.

### Sample size estimation

Data in the literature indicated that the proximal colon AMR in the CO_2_ group would be 33% [3]. To show a clinically important improvement in the proximal colon AMR reduction by WE colonoscopy, we assumed that WE should reduce the AMR to 15% compared with previously reported data. With a statistical power of 80% and a two-sided significance level of 0.05, 85 patients were needed in each arm of the comparison.

### Statistical analysis

Categorical and continuous variables were summarized as frequencies and percentages and as mean with standard deviation (SD), respectively. Student’s *t*-test for continuous factors, the Wilcoxon rank sum test for ordinal variables, and the Chi-square test for categorical variables were used to assess differences in the demographic and clinical characteristics. All analyses were performed using SAS version 9.3 or later (SAS Institute Inc., Cary, NC, USA). A *P* value < 0.05 was considered significant.

## Results

**Fig. 1:**
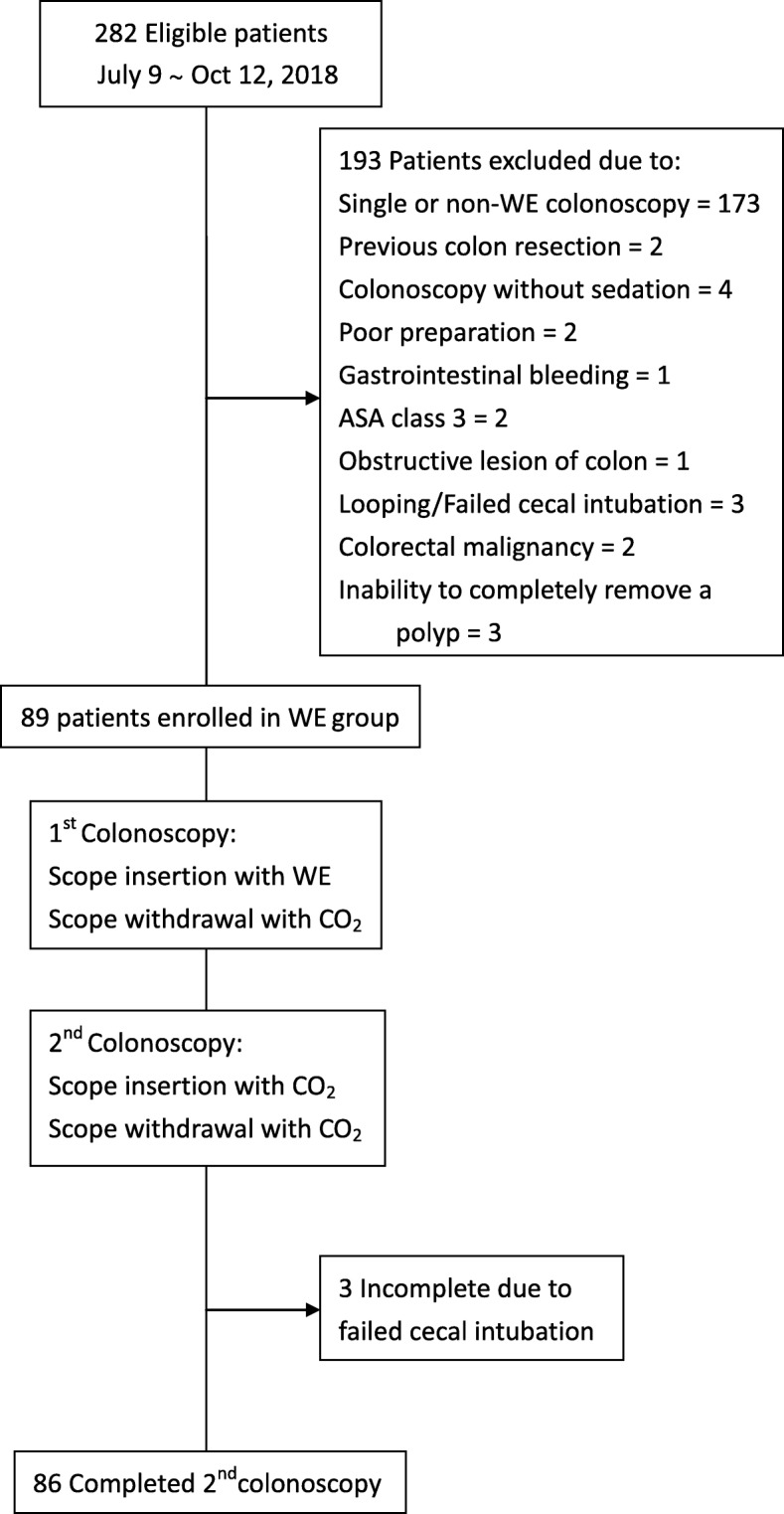
Flow chart of study 1 (the WE group). Abbreviation: *ASA* American Society of Anesthesiology, *WE* water exchange.

**Fig. 2:**
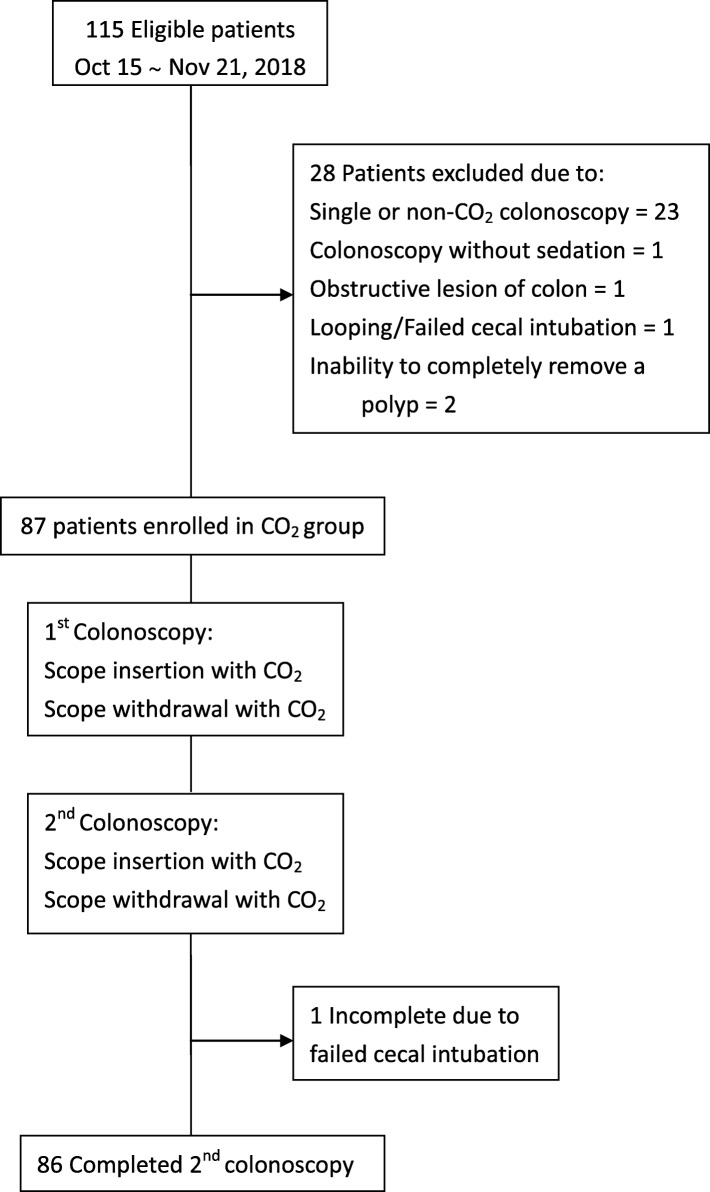
Flow chart of study 2 (the CO_2_ group)

The study flow charts are shown in Figs. 1 and 2. In study 1 (the WE group), WE directed at near-complete removal netted 89% of infused water upon arrival to the cecum. WE showed considerably lower AMRs in the right (17.5%) and proximal (15.5%) colon than those in the literature (22.5–27% in the right and 23.4–33.3% in the proximal colon) [2, 3]. In study 2 (the CO_2_ group), the AMRs in the CO_2_ group were 33.8% (right colon) and 30.4% (proximal colon).

The demographic variables were comparable except that a significantly higher proportion of patients (61.6%) underwent colonoscopy for surveillance in the WE group, while a significantly higher proportion of patients (55.8%) underwent colonoscopy for screening in the CO_2_ group (Table 1).

**Table 1:** Demographics details and indications for colonoscopy

Variable	Study 1 (WE group) (*n* = 86)	Study 2 (CO_2_ group) (*n* = 86)	*P* value
Male gender, n (%)	43 (50.0)	47 (54.7)	0.647
Age, mean (SD), years	53.4 (10.7)	52.9 (9.6)	0.747
Body weight, mean (SD), kg	66.8 (14.1)	67.7 (12.4)	0.649
Body mass index, mean (SD), kg/m^2^	25.2 (4.3)	24.7 (3.3)	0.478
Previous abdominal surgery, n (%)	30 (34.9)	24 (27.9)	0.412
Active smoker, n (%)	18 (20.9)	13 (15.1)	0.428
Family history of CRC in first degree relative, n (%)	3 (3.5)	7 (8.1)	0.329
Indications for colonoscopy, n (%)			0.032
Screening	33 (38.4)	48 (55.8)	
Surveillance	53 (61.6)	38 (44.2)	

Abbreviation: *WE* Water exchange, *SD* standard deviation, *CRC* colorectal cancer

**Table 2:** Colonoscopy procedural data

Variable	WE group (*n* = 86)	CO_2_ group (*n* = 86)	*P* value
First-pass insertion time, mean (SD), minutes	8.65 (3.01)	6.18 (4.59)	< 0.0001
First-pass withdrawal time, mean (SD), minutes	17.85 (7.71)	21.00 (7.50)	0.007
First-pass total procedure time, mean (SD), minutes	26.51 (8.52)	27.18 (8.56)	0.605
Second-pass insertion time, mean (SD), minutes	3.33 (1.66)	3.92 (2.42)	0.062
Second-pass withdrawal time, mean (SD), minutes	9.06 (2.91)	10.44 (3.37)	0.005
Second-pass total procedure time, mean (SD), minutes	12.39 (3.43)	14.36 (4.38)	0.001
Boston Bowel Preparation Scale score^a^, mean (SD)	7.4 (0.7)	7.0 (0.5)	< 0.0001
Water infused during insertion^a^, mean (SD), mL	1513.8 (510.5)	43.0 (79.7)	< 0.0001
Water aspirated during insertion^a^, mean (SD), mL	1341.3 (437.0)	122.7 (108.8)	< 0.0001
Aspirated as percent of infused during insertion^a^, mean (SD), %	89.3 (9.5)	NA	NA
Water infused during withdrawal^a^, mean (SD), mL	261.5 (387.1)	587.9 (341.0)	< 0.0001
Water aspirated during withdrawal^a^, mean (SD), mL	358.2 (332.8)	556.0 (297.9)	< 0.0001
Need for position change^a^, n (%)	48 (55.8)	45 (52.3)	0.760
Need for abdominal compression^a^, n (%)	52 (60.5)	74 (86.0)	0.0002
In patients undergoing colonoscopy only, n (%)	57 (66.3)	53 (61.6)	0.634
Fentanyl dose during first-pass examination, mean (SD), ug	73.9 (16.9)	72.4 (13.6)	0.610
Midazolam dose during first-pass examination, mean (SD), mg	4.25 (1.13)	4.53 (1.14)	0.209
Total fentanyl dose during first- and second-pass examinations, mean (SD), ug	82.5 (17.1)	79.3 (16.5)	0.335
Total midazolam dose during first- and second-pass examinations, mean (SD), mg	4.69 (1.11)	4.89 (1.23)	0.388

^a^ Data were referred to first-pass examination only

Abbreviation: *WE* Water exchange, *SD* standard deviation, *NA* not applicable

Table 2 details the procedural outcomes. For the first-pass examination, the insertion time and infused water volume during insertion in the WE group were significantly longer and greater, respectively, than those in the CO_2_ group. The withdrawal time and infused water volume during withdrawal in the CO_2_ group were significantly longer and higher, respectively, than those in the WE group. The total procedure time of the first-pass examination was comparable between the two studies. However, the withdrawal time and total procedure time of the second-pass examination were significantly longer in the CO_2_ group. The cleansing scores were significantly higher in the WE group. Compared with the CO_2_ group, a significantly lower portion of participants in the WE group required abdominal compression to reach the cecum. There was no significant difference between the two studies with respect to the doses of sedative agents in patients undergoing colonoscopy alone. There were no adverse events in either study.

The detection of adenoma and proximal hyperplastic polyps is reported in Table 3. There was no significant difference in the overall ADR or for adenomas in any location between the two studies. Similarly, the overall MAP, right colon MAP, and proximal colon MAP did not differ statistically between the two studies. The overall and proximal colon advanced ADRs were also comparable. There was no significant difference in the PHP-DR between the two studies.

**Table 3:** Detection of adenoma and proximal colon hyperplastic polyp during first-pass examination

Variable	WE group (*n* = 86)	CO_2_ group (*n* = 86)	*P* value
Overall ADR, n (%) [95% CI]	46 (53.5) [42.4, 64.3]	50 (58.1) [47.0, 68.7]	0.645
ADR for screening indication, n (%) [95% CI]	16/33 (48.5) [30.8, 66.5]	30/48 (62.5) [47.4, 76.0]	0.257
ADR for surveillance indication, n (%) [95% CI]	30/53 (56.6) [42.3, 70.2]	20/38 (52.6) [35.8, 69.0]	0.831
Right colon ADR, n (%) [95% CI]	32 (37.2) [27.0, 48.3]	30 (34.9) [24.9, 45.9]	0.874
Proximal colon ADR, n (%) [95% CI]	39 (45.3) [34.6, 56.5]	38 (44.2) [33.5, 55.3]	1.000
Distal colon ADR, n (%) [95% CI]	22 (25.6) [16.8, 36.1]	27 (31.4) [21.8, 42.3]	0.499
Overall advanced ADR, n (%) [95% CI]	5 (5.8) [1.9, 13.1]	5 (5.8) [1.9, 13.1]	1.000
Proximal colon advanced ADR, n (%) [95% CI]	2 (2.3) [0.3, 8.2]	2 (2.3) [0.3, 8.2]	1.000
Overall MAP (SD)	1.2 (1.4)	1.4 (1.8)	0.394
Right colon MAP (SD)	0.6 (1.0)	0.6 (1.1)	0.827
Proximal colon MAP (SD)	0.8 (1.2)	0.9 (1.4)	0.680
Right colon mean number of adenoma and HP (SD)	0.9 (1.1)	0.8 (1.1)	0.739
Proximal colon mean number of adenoma and HP (SD)	1.3 (1.5)	1.2 (1.4)	0.797
PHP-DR, n (%) [95% CI]	23 (26.7) [17.8, 37.4]	26 (30.2) [20.8, 41.1]	0.736

Eight and seven patients in the WE group and CO_2_ group had adenoma found only during the second-pass examination; and the ADR for the combined examinations would be 62.8% (95% CI, 51.7–73.0%) for WE, and 66.3% (95% CI, 55.3–76.1%) for CO_2_, respectively (*P* = 0.75)

Abbreviation: *WE* Water exchange, *ADR* adenoma detection rate, *CI* confidence interval, *MAP* mean adenoma per procedure, *SD* standard deviation, *HP* hyperplastic polyp, *PHP-DR* proximal hyperplastic polyp detection rate

The per-participant miss rates for adenoma and proximal hyperplastic polyps are reported in Table 4. There were no significant differences in the miss rates between the WE and CO_2_ groups for any adenoma, right colon adenoma, distal colon adenoma, or right and proximal colon hyperplastic polyps. Compared with the WE group, the CO_2_ group showed significantly higher per-participant miss rates for proximal colon adenoma, as well as for right and proximal colon combined adenoma and hyperplastic polyps.

**Table 4:** Miss rates per participants (number of participants with adenoma/hyperplastic polyp missed in first-pass examination as indicated by column head)

Miss rate	WE group (*n* = 86)	CO_2_ group (*n* = 86)	*P* value
Any adenoma, n (%) [95% CI]	19 (22.1) [13.9, 32.3]	27 (31.4) [21.8, 42.3]	0.228
Diminutive (≤5 mm) adenoma, n (%) [95% CI]	15 (17.4) [10.1, 27.1]	25 (29.1) [19.8, 39.9]	0.104
Right colon adenoma, n (%) [95% CI]	10 (11.6) [5.7, 20.4]	19 (22.1) [13.9, 32.3]	0.102
Proximal colon adenoma, n (%) [95% CI]	12 (14.0) [7.4, 23.1]	24 (27.9) [18.8, 38.6]	0.038
Distal colon adenoma, n (%) [95% CI]	9 (10.5) [4.9, 18.9]	9 (10.5) [4.9, 18.9]	1.000
Right colon hyperplastic polyp, n (%) [95% CI]	1 (1.2) [0.03, 6.3]	7 (8.1) [3.3, 16.1]	0.064
Proximal colon hyperplastic polyp, n (%) [95% CI]	6 (7.0) [2.6, 14.6]	11 (12.8) [6.6, 21.7]	0.307
Right colon combined adenoma and hyperplastic polyp, n (%) [95% CI]	10 (11.6) [5.7, 20.4]	24 (27.9) [18.8, 38.6]	0.012
Proximal colon combined adenoma and hyperplastic polyp, n (%) [95% CI]	16 (18.6) [11.0, 28.5]	33 (38.4) [28.1, 49.5]	0.007

Abbreviation: *WE* Water exchange, *CI* confidence interval

The per-adenoma/polyp AMR and HPMR details are presented in Table 5. Compared with the CO_2_ group, the WE group showed significantly lower AMR (17.5% vs. 33.8% [right colon], *P* = 0.034; 15.5% vs. 30.4% [proximal colon], *P* = 0.018) and combined AMR and HPMR (13.6% vs. 32.4% [right colon], *P* = 0.002; 15.2% vs. 30.5% [proximal colon], *P* = 0.003). There was no significant difference in the AMR of the distal colon (26.3% [WE group] vs. 23.5% [CO_2_ group], *P* = 0.81). According to the United States Multi-Society Task Force on Colorectal Cancer guidelines for colonoscopy surveillance after screening and polypectomy, a significant change in the postpolypectomy surveillance schedule was indicated by the second examination in 15 patients (8 patients in the WE group vs. 7 patients in the CO_2_ group, *P* = 1.00) [20].

**Table 5:** Miss rates per adenoma/polyp (number of adenomas/hyperplastic polyps missed in first colonoscopy as indicated by column head)

Miss rate	WE group	CO_2_ group	*P* value
Overall AMR, n/N (%) [95% CI]	23/122 (18.9) [12.3, 26.9]	46/163 (28.2) [21.5, 35.8]	0.071
Size category, n/N (%) [95% CI]			0.069
≤5 mm	18/94 (19.1) [11.8, 28.6]	35/128 (27.3) [19.8, 35.9]	0.202
6–9 mm	4/24 (16.7) [4.7, 37.4]	9/28 (32.1) [15.9, 52.4]	0.336
≥10 mm	1/4 (25.0) [0.6, 80.6]	2/7 (28.6) [3.7, 71.0]	1.000
Right colon AMR, n/N (%) [95% CI]	11/63 (17.5) [9.1, 29.1]	25/74 (33.8) [23.2, 45.7]	0.034
Size category, n/N (%) [95% CI]			0.022
≤5 mm	9/50 (18.0) [8.6, 31.4]	20/65 (30.8) [19.9, 43.5]	0.134
6-9mm	2/12 (16.7) [2.1, 48.4]	4/6 (66.7) [22.3, 95.7]	0.107
≥10 mm	0/1 (0.0) [0.0, 97.5]	1/3 (33.3) [0.8, 90.6]	1.000
Right colon HPMR, n/N (%) [95% CI]	1/25 (4.0) [0.1, 20.4]	9/31 (29.0) [14.2, 48.0]	0.031
Right colon combined AMR and HPMR, n/N (%) [95% CI]	12/88 (13.6) [7.3, 22.6]	34/105 (32.4) [23.6, 42.2]	0.002
Proximal colon AMR, n/N (%) [95% CI]	13/84 (15.5) [8.5, 25.0]	34/112 (30.4) [22.0, 39.8]	0.018
Size category, n (%) [95% CI]			0.019
≤5 mm	11/68 (16.2) [8.4, 27.1]	27/92 (29.3) [20.3, 39.8]	0.062
6–9 mm	2/15 (13.3) [1.7, 40.5]	5/16 (31.3) [11.0, 58.7]	0.394
≥10 mm	0/1 (0.0) [0.0, 97.5]	2/4 (50.0) [6.8, 93.2]	1.000
Proximal colon HPMR, n/N (%) [95% CI]	7/48 (14.6) [6.1, 27.8]	13/42 (31.0) [17.6, 47.1]	0.078
Proximal colon combined AMR and HPMR, n/N (%) [95% CI]	20/132 (15.2) [9.5, 22.4]	47/154 (30.5) [23.4, 38.4]	0.003
Distal colon AMR, n/N (%) [95% CI]	10/38 (26.3) [13.4, 43.1]	12/51 (23.5) [12.8, 37.5]	0.807

Abbreviation: *WE* Water exchange, *AMR* adenoma miss rate, *CI* confidence interval, *HPMR* hyperplastic polyp miss rate

## Discussion

Study 1 showed WE considerably lowered AMRs in the right and proximal colon compared with data in the literature. Study 2 found that compared with CO_2_ insufflation, WE significantly reduced the right and proximal colon AMRs in our hands (16 and 15%), respectively. Furthermore, WE significantly decreased the combined AMR and HPMR in the right and proximal colon (19 and 15%), respectively.

In a meta-analysis of 17 RCTs with 10,350 patients, WE achieved a significantly higher overall ADR than air (OR, 1.40; 95% credible interval [CrI], 1.22 to 1.62), or CO_2_ (OR, 1.48; 95% CrI, 1.15 to 1.86) insufflation [18]. WE achieved the highest ADR in the right colon and in screening cases. Improved bowel cleanliness and reduced withdrawal cleaning-related multitasking distractions were plausible mechanisms by which WE improved ADR [18, 21]. These advantages of WE may translate into reduced AMRs. The impact of WE with near-complete removal of infused water during insertion on AMR, however, had not been reported.

In a meta-analysis of 43 tandem colonoscopy studies, the pooled per-adenoma AMR was 26% (95% CI, 23–30%), and the AMR by size was 6% (95% CI: 2–11%) for large adenomas, 17% (95% CI, 11–24%) for small adenomas, and 28% (95% CI: 23–34%) for diminutive adenomas [6]. Additionally, a right colon AMR of 22.5 to 27% and a proximal colon AMR of 23.4 to 33.3% have been reported [2–6]. The lower AMR (15.5%, proximal colon) of WE compared with that in previous reports prompted us to evaluate the AMR of CO_2_ insufflation. In study 2, the overall AMR and the AMRs in the right and proximal colon in the CO_2_ group were similar to those in previous reports. In our studies, the AMRs in the right colon (17.5% [WE] vs. 33.8% [CO_2_]) and proximal colon (15.5% [WE] vs. 30.4% [CO_2_]) were significantly lower (both *P* < 0.05) in the WE group, suggesting that WE increased effectiveness by lowering the AMRs compared with those of CO_2_ insufflation. The hypotheses supporting our observation that WE decreased right and proximal colon AMRs compared with CO_2_ insufflation comprised of facilitation of cecal intubation, improvement of right colon cleanliness, increased ADR in the right and proximal colon, and reduction of cleaning-related multitasking distractions during withdrawal, all by WE.

The results in the current report were in contrast with a recent study of total underwater colonoscopy versus CO_2_ insufflation [22]. In that study, the authors hypothesized that water was a superior medium to gas for visualizing the mucosa, and they filled the lumen with water during withdrawal in addition to water infusion during insertion. The results showed that total underwater colonoscopy had a significantly higher overall AMR than colonoscopy performed with CO_2_ insufflation (36% vs. 23%, *P* = 0.025). The discrepancy could be explained by the proportion of infused water removed during insertion, i.e., 66% (moderate) in the total underwater colonoscopy study and 89% (near-complete) in the current study. In previous RCTs showing that WE significantly increased the ADR, 91 to 102% of infused water was suctioned upon arrival to the cecum [14–17]. With limited water and debris removal, optimal salvage cleaning could not be achieved during insertion and the endoscopists using total underwater colonoscopy had impaired visualization under water during withdrawal, possibly accounting for the higher miss rates.

Our study has some limitations. First, this investigation consisted of two consecutive observational studies with unblinded examiners, not a RCT with blinded colonoscopists. Second, the significant difference in the indications for colonoscopy between the WE and CO_2_ groups could have introduced confounding issues. Third, there was a significant difference in the withdrawal times of the first- and second-pass examinations. In the WE group, the possibility that the examiner exerted less effort reflected by the shorter withdrawal time to detect fewer lesions could not be excluded. In the CO_2_ group, the increased withdrawal time of the first-pass and second-pass examinations was likely attributed to the additional time for mucosal cleaning, and more polyps removed, respectively. Our study has some strength. The high ADR in both groups (> twice the recommended standard) attested to the high quality of the examinations. The results in this report are important observations that will provide data for sample size calculation in a RCT.

## Conclusions

In conclusion, in contrast to total underwater colonoscopy (with insertion suction of only 66% of infused water), which increased the AMR**,** WE with near-complete (89%) removal of infused water during insertion significantly decreased the AMRs and combined AMR and HPMR in the right and proximal colon compared with those of CO_2_ insufflation. The provocative data warrant confirmation in a RCT.

## Data Availability

All data used and/or analyzed during the current study are included in this published article and are also available from the corresponding author on reasonable request.

## Abbreviations

ADR: Adenoma detection rate
AMR: Adenoma miss rate
BBPS: Boston Bowel Preparation Scale
CI: Confidence interval
CO_2_: Carbon dioxide
CRC: Colorectal cancer
Crl: Credible interval
HPMR: Hyperplastic polyp miss rate
MAP: Mean adenoma per procedure
OR: Odds ratio
PCCRC: Postcolonoscopy colorectal cancer
RCT: Randomized controlled trial
SD: Standard deviation
WE: Water exchange
